# Association between physical activity and physical and functional performance in non-institutionalized Mexican older adults: a cohort study

**DOI:** 10.1186/s12877-022-03083-7

**Published:** 2022-05-03

**Authors:** Brenda María Martínez-Hernández, Oscar Rosas-Carrasco, Miriam López-Teros, Alejandra González-Rocha, Paloma Muñoz-Aguirre, Rosa Palazuelos-González, Araceli Ortíz-Rodríguez, Armando Luna-López, Edgar Denova-Gutiérrez

**Affiliations:** 1 Facultad de Medicina, Universidad Nacional Autónoma de México, Ciudad de Mexico, México; 2 Departamento de Salud, Universidad Iberoamericana, Ciudad de Mexico, México; 3 Centro de Investigación en Nutrición y Salud, Instituto Nacional de Salud Pública, Cuernavaca, México; 4 CONACYT-Centro de Investigación en Salud Poblacional, Instituto Nacional de Salud Pública, Cuernavaca, México; 5 Center for Evaluation and Survey Research, National Institute of Public Health, Cuernavaca, Mexico; 6 Facultad de Nutrición, Universidad Autónoma del Estado de Morelos, Cuernavaca, México; 7 Instituto Nacional de Geriatría, Ciudad de Mexico, México

**Keywords:** Physical activity, Activities of Daily Living, Physical Performance, Older adults

## Abstract

**Background:**

Aging is an independent risk factor for deterioration in functional capacity. Some studies have reported that physical activity (PA) improves functional capacity and physical performance among older adults (OA). Thus the objective of the present study was to assess the longitudinal association between PA and functional and physical performance in non-institutionalized OA.

**Methods:**

A longitudinal analysis using data from the Frailty, Dynapenia and Sarcopenia in Mexican adults (FRADYSMEX, by its Spanish acronym) cohort study was conducted. PA was assessed through the Community Healthy Activities Model Program for Seniors (CHAMPS) instrument. Functionality was measured with the Barthel index and the Lawton and Brody scale, while physical performance was measured with the Short Physical Performance Battery (SPPB). To evaluate the association between the level of PA and physical and functional performance as a continuous variable, a linear regression of mixed effects was performed. To assess PA and dependence in basic activities of the daily life (BADL), instrumental activities of the daily life (IADL), and low physical performance (PP), generalized estimation equation models [to compute odds ratios (OR) and 95% confidence intervals (95%CI)] were computed.

**Results:**

Older people who performed moderate to vigorous-intensity PA had a lower risk of dependence in IADL (OR = 0.17; 95%CI: 0.10, 0.80) and lower risk of low PP (OR = 0.18; 95%CI: 0.11, 0.58) compared to those in lower categories of PA.

**Conclusions:**

Older adults living in the community who perform PA of moderate to vigorous intensity have a lower risk of dependence in BADL and IADL and have a lower risk of low PP.

## Background

Aging is a multifactorial phenomenon, which is characterized by the accumulation of degenerative processes that arise from molecular alterations and damage that compromise tissue and cellular functions [1]. It is an independent risk factor for the development of non-communicable diseases and it also increases the probability of deterioration in functional capacity, whether physical or mental [2]. During this period, various systems undergo physiological and morphological alterations that can negatively influence physical capacity, leading to difficulties in carrying out activities of daily living (ADL) [3].

The increasing prevalence of chronic diseases constitutes a particular challenge for older adults (OA) as it affects functionality, generates disability, and eventually leads to dependency. Therefore, the importance of OA’s functional status must be recognized, since it largely determines the relationship between population aging and health spending [4]. Regarding loss of autonomy, a previous study [5] conducted in the Mexican population showed that 26.9% of OA exhibit some degree of limitation in basic activities of daily living (BADL). These data show that functional dependence is one of the most serious problems experienced by OA, since it hinders care and access to health services and is particularly disruptive when accompanied by cognitive impairment.

In order to preserve their physical capacities and avoid developing ADL-dependency, older adults must adopt healthy lifestyles throughout their lives [6]. These include physical activity (PA), such as walking, cycling, housework and gardening [7], and eating an adequate diet. Moderate to vigorous PA has been associated with multiple benefits: physiological (i.e. increases strength, improves oxygen consumption, leads to cardiovascular and metabolic adaptations) [8, 9]; psychological (i.e. improves mood, reduces stress and anxiety) [10], cognitive (e.g. executive functions); and social [11]. These help to maintain an optimal intrinsic capacity and therefore improve functional capacity [12].

Some longitudinal studies have reported that older people who perform PA of high to moderate intensity are associated with a better ability to perform their basic and instrumental activities of daily living [13–16]. However, to our knowledge, not longitudinal studies have assessed jointly physical performance and it is association with the BADL, few studies from other design asses this relationship in adults older of 60 years [14, 16, 17].

In Mexico, only one study has evaluated the relationship between PA and disability, in the short-term [18]. However, there is no evidence related to PA and physical performance. For that reason, the study could support future recommendations for community dwelling older adults for improve their functionality and independence, that would translate in better quality of life.

Therefore, the aim of the present study was to assess the association between PA and physical and functional performance in non-institutionalized OA in Mexico.

## Methods

### Design and study population

A secondary analysis using data from the cohort titled Frailty, Dynapenia, and Sarcopenia in Mexican Adults (FraDySMex) [19–21] was conducted. The first measurement was carried out from October 2014 to December 2015 and the second was carried out from October to December 2019.

The study was approved by the Ethics and Research Committee of the Hospital Ángeles Mocel and registered by the National Institute of Geriatrics (Protocol number: DI-PI-002/2014). The study was carried out considering the principles established in the Declaration of Helsinki, the international ethical guidelines for biomedical research in human beings, and the provisions of the Official Mexican Standard NOM-012-SSA3-2012. Finally, informed consent was given in writing to the participants prior to the study.

### Participants

The adults were invited to participate through home visits, which were done by psychologists and social workers. Additionally, announcements were left in churches, community, social security and health centers. The people eligible to participate in this cohort were women and men adults > 50 years old and those who: Were able to walk with or without a gait aid; those who answered the study questionnaires on their own or with the help of a caregiver; those with a score of ≤ 10 on the Mini-mental state examination [22]; and those who were able to complete the physical tests. For the present analysis, only the OA ≥ 60 years were included. The exclusion criteria were: Institutionalization and any condition that the clinical staff deemed could affect the individual’s ability to answer the questionnaires or to complete the physical tests. The elimination criteria included files that did not have complete information on the participants. Assessments were performed by trained medical personnel, composed of geriatricians, internists, general practitioners, nurses, physiotherapists, nutritionists and rehabilitation specialists, and conducted in the Research Laboratory of Functional Assessment of the National Institute of Geriatrics in Mexico and the Iberoamerican University. The selection criteria for participants in this cohort (FraDySMex) were mainly due to the study required participants who could attend the assessment centers with or without assistance. It was necessary for the objective evaluation of body composition with the dual X-ray absorptiometry equipment and muscle strength by dynamometry necessary for the diagnosis of osteoporosis, dynapenia, frailty and sarcopenia, the main study conditions of this cohort.

**Fig. 1:**
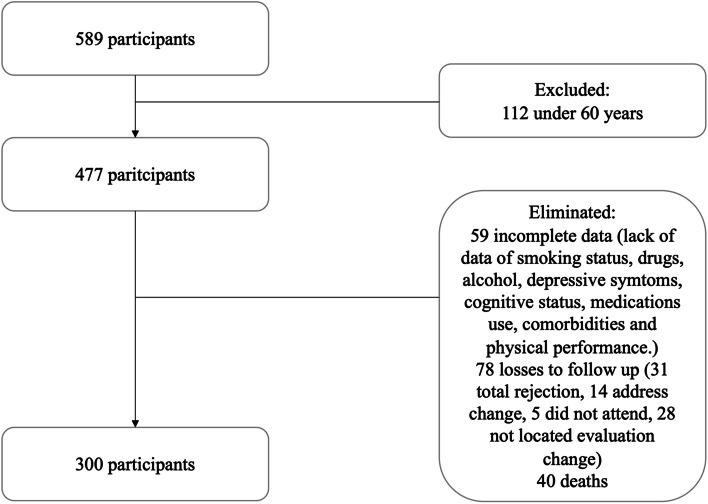
Flowchart of the study population

Initially, a total of 589 participants were included in FraDySMex. For the present analysis, 112 were excluded because they were under 60 years of age; in addition, 177 data were eliminated due to loss to follow-up and 59 did not have complete data. For the final analysis, 300 individuals were included (Fig. 1). In general, the excluded OA were similar to those included in the final analysis; for example, they were predominantly women, married, and had similar average years of schooling.

### Functional performance assessment

We used the Barthel Index and the Lawton and Brody Scale to evaluate functionality.

#### Basic activities of daily living assessment

The Barthel Index (BI) is an instrument that measures the capacity of a person to carry out ten Activities of Daily Living (ADL), considered as basic, obtaining a quantitative estimation of its degree of independence [23]. The activities of daily living that it evaluates are: Feeding, transfer from chair to bed, bathing, dressing, grooming, stool, urination, use of the toilet, walking and climbing stairs. The items of the BI possess a hierarchy of difficulty and yield ordinal intervals between adjacent scores, the total score ranges from 0, indicating that they are completely dependent, to 100, completely independent [23, 24]. The reliability of the instrument applied to older people living in the community, measured with Cronbach’s α, is 0.82, which indicates good reliability [25].

On the other hand, Barthel index also is a validated scale for international use to assess functional capacity in older adults as Katz scale evaluated the structural and predictive validity, internal consistency, sensitivity to change of the Barthel Index scores and their relationship with other measurement instruments through 4 cohorts. The factor loadings were greater than 0.40, and the fit indices were satisfactory. The internal consistency of the scores was optimal and the results confirm the one-dimensional structure found in other validation studies of the original version. The predictive validity of their scores regarding mortality at 6 months, the cut-off value of 95 for both cohorts showed an area under the curve (AUC) of 0.742 [26–30].

#### Instrumental activities of daily living assessment

The Lawton and Brody scale measures the instrumental activities of daily living (IADL), which cover eight domains, including: Ability to use the telephone, use of transportation, medications, finances, food preparation, shopping, home care, and laundry [31]. The score for each item ranges from 0 to 1 and is related to the performance of the activity. The total score ranges from 0 (maximum dependence) to 8 (total independence) [31, 32]. Although a diversity of questionnaires has been developed to address these constructs, the most broadly used in research setting to examine BADLs is the Barthel Index, and for IADLs, the Lawton and Brody scale. As for the case of the IADLs, they have been commonly assessed with the Lawton and Brody scales, a scale that has been shown to be suitable for application in community dwelling OA, but also in hospital, short-term care, and rehabilitation facilities [32]. Additionally, it presents a Cronbach alfa of 0.94 that represents high reliability and highs sensitivity for the change [33, 34]. This instrument is reliable for evaluating instrumental daily living activities in older adults living in the community and it has been previously validated in Spanish [34].

### Physical Performance assessment

Physical performance was measured through the Short Physical Performance Battery (SPPB) which consists of three subtests. The first tests balance, in which the participants are asked to stand while keeping their feet together, then stand in a semi-tandem position, and finally in tandem. Each position must be held for 10 s. The next test is gait speed, where the person has to walk at their usual pace for four meters; the time it took them to walk the path is measured. The last subtest is to get up five consecutive times from a chair; the person is asked to do it as quickly as possible with their arms crossed over their chest [35]. Each of the subtests is assigned a score ranging from 0 to 4. Obtaining a final score of 12 points indicates that the person has a good physical performance and a score less than 8 shows low physical performance. This instrument has been previously validated in Spanish [35]. Additionally, previous reports identify this tool with good accuracy for physical performance [36].

### Physical Activity assessment

The Community Healthy Activities Model Program for Seniors (CHAMPS) questionnaire was used to assess the PA of OA. This questionnaire consists of 41 activities of low to vigorous intensity and the questions require older people to report the number of times and the approximate duration in hours per week in the last 4 weeks that they did some of the questionnaire activities. A metabolic equivalents (METs) value was assigned to each activity, which was adjusted according to the activity. The measures that can be obtained from the questionnaire are caloric expenditure per week, METs, hours and frequency of PA per week. This questionnaire was adapted and translated for Latino OA [37]. This tool could identify different types of exercise and levels of intensity more suitable for the OA [38, 39].

Longitudinal PA data were acquired at baseline and 4–5 years later. In the present study persons were classified into one of three PA groups: 1) those who met the PA guidelines (≥ 6, moderate to vigorous, METs), 2) insufficiently increased PA (≥ 3.0 < 6.0, moderate to vigorous, METs), or 3) those who do not increase moderate to vigorous PA or remained inactive.

### Covariables

A questionnaire was applied to the participants to obtain sociodemographic information like age, occupation (e.g. unemployed, retired with a pension, retired without a pension, informal business, day laborer, worker, office worker, independent professional, employer/boss/entrepreneur, homemaker, inactive or other), marital status, and years of schooling.

For use of medication, participants were asked the name of the medicine, the pharmaceutical form, the frequency, the time of use, if it was medically prescribed, and the route of administration. Data on current or previous smoking habits, frequency of alcohol consumption, and use of drugs were also investigated.

Nutritional status was evaluated through the Mini Nutritional Assessment (MNA), which assesses the risk of malnutrition in older people. Scores with < 17 indicated protein-calorie malnutrition, 17 to 23.5 risk of malnutrition, and ≥ 24 good nutritional status [40].

Cognitive level was measured through the Mini-mental state examination, which consists of 30 items and assesses cognitive functions such as orientation, attention, calculation, memory, language, verbal and written comprehension, reading, writing and constructional skills [22].

The Charlson comorbidity index was used as a prognostic instrument for comorbidities. This index consists of 19 medical conditions; each one is assigned a weighted score according to the relative risk of mortality at 1 year and the total score is the sum of all the clinical entities that the patient presents [41, 42].

The shortened version of the Center for Epidemiological Studies Depression Scale (CESD-7) was used to assess the presence of depression symptoms [43]. This instrument has been previously validated in Mexican OA [44].

### Statistical analysis

The normality of the data distribution was corroborated using the Kolmogorov–Smirnov test. A descriptive analysis of the main variables of interest was performed. Means and standard deviation were used when the variables were quantitative and presented a normal distribution; otherwise, geometric means and confidence intervals were used. For the qualitative variables, frequencies were obtained.

To evaluate the association between the level of PA and physical and functional performance as a continuous variable, a linear regression of mixed effects model was performed. To assess PA and dependence in BADL, IADL and low PP, generalized estimation equation models were computed; both models were adjusted for the covariates. Based on the biological plausibility and the previous literature, these adjustment variables were included [16, 45–47].

All *P* values < 0.05 were considered statistically significant. The statistical package used for the analysis was STATA for windows, version 14.0.

## Results

The mean age of the participants was 71.3 (±7.9) years and 83% of the participants were women. The mean educational level was 9.1 years, 63.8% women were homemakers and 60.7% of the men were retired with a pension, while 78.5% of the participants had a good nutritional status. OA had a mean score in CESD of 4.3 (95% CI 3.9–4.8), being higher in women than in men (4.4 vs 3.9; *P* = 0.003). Regarding the scores in BADL, IADL, and physical performance, no significant differences were found between men and women (Table 1).

**Table 1:** Sociodemographic characteristics stratified by sex

	Total *n* = 300 mean ^a^ (95% CI)	Men *n* = 51 mean ^a^ (95% CI)	Women *n* = 249 mean ^a^ (95% CI)	*P* value
Age (years), mean (SD)	71.3 (7.9)	71.5 ± 7.5	71.3 ± 8.0	0.887 ^b^
Marital status, n (%)				
Married	111(37.1)	35 (68.6)	76 (30.6)	< 0.001^c^
Free Union	4 (1.3)	3 (5.8)	2 (0.4)	
Single	46 (15.3)	4 (7.8)	42 (16.9)	
Separated	13 (4.3)	0(0)	13 (5.2)	
Divorced	19 (6.3)	2(3.9)	17 (6.8)	
Widowed	106 (35.4)	7 (13.7)	99 (39.9)	
Years of schooling	9.1 (8.6–9.74)	11.0 (9.7–12.4)	8.8 (8.2–9.4)	0.001^d^
Occupation, n (%)				
Unemployed	2 (0.6)	2 (3.9)	0(0)	< 0.001^c^
Retiree with pension	83 (27.6)	31 (60.7)	52 (20.8)	
Retiree without pension	13 (4.3)	7 (13.7)	6 (2.4)	
Informal commerce	6 (2.0)	2 (3.9)	4 (1.6)	
Office worker	10 (3.3)	1 (1.9)	9 (3.6)	
Independent professional	10 (3.3)	3 (5.8)	7 (2.8)	
Businessman or boss	3 (1.0)	2 (3.9)	1 (0.4)	
Housewife	159 (53.0)	0 (0)	159 (63.8)	
Inactive	2 (0.6)	1 (1.9)	1 (0.4)	
Other activity	12 (4.0)	2 (3.9)	10 (4.0)	
Nutrition status, n (%)				
Normal	227 (78.5)	45 (88.2)	182 (76.47)	0.176 ^e^
Risk of malnutrition	59 (20.4)	6 (11.7)	53 (22.2)	
Malnutrition	3 (1.0)	0(0)	3 (1.2)	
Smoking status, n (%)				
Current tobacco use	38 (12.6)	8 (15.6)	30 (12.0)	0.034 ^e^
Currently does not consume	102 (34.0)	24 (47.0)	78 (31.3)	
Never	160 (53.3)	19 (37.2)	141 (56.6)	
Drugs, n (%)				
Consume	111 (37.3)	14 (27.4)	97 (39.4)	0.115 ^e^
Does not consume	186 (62.6)	37 (72.5)	149 (60.5)	
Alcohol, n (%)				
Has never drunk alcohol	88 (29.4)	8 (15.6)	80 (32.2)	< 0.001^c^
Currently does not drink	51 (17.0)	12 (23.5)	39 (15.7)	
Drink sporadically	138 (46.1)	19 (37.2)	119 (47.9)	
Less than once a week	13 (4.3)	7 (13.7)	6 (2.4)	
One or two days a week	3 (1.0)	3 (5.8)	0(0)	
Five to six days a week	1 (0.3)	0(0)	1 (0.4)	
Every day of the week	5 (1.6)	2 (3.9)	3 (1.2)	
Depressive symptoms	4.3 (3.9–4.8)	3.9 (2.9–5.3)	4.4 (3.9–5.0)	0.003 ^d^
Cognitive status	26.6 (26.3–27.0)	27.4 (26.7–28.1)	26.5 (26.1–26.9)	0.038 ^d^
Medication use	3.9 (3.6–4.3)	3.3 (2.6–4.1)	4.1 (3.8–4.5)	0.038 ^d^
Presence of comorbidities	1.6 (1.5–1.8)	1.7 (1.4–2.1)	1.6 (1.5–1.8)	0.688 ^d^
BADL^f^	98.4 (98.0, 98.9)	98.1 (97.0, 99.2)	98.5 (98.0, 99.0)	0.252 ^d^
IADL^g^	7.6 (7.5, 7.7)	7.5 (7.3, 7.8)	7.6 (7.5, 7.7)	0.106 ^d^
Physical performance	8.7 (8.4,9.0)	9.4 (9.0, 9.8)	8.5 (8.2, 8.8)	0.037 ^d^
Moderate to vigorous PA^h^	2.2 (1.9, 2.5)	2.9 (2.1, 4.1)	2.1 (1.8, 2.4)	0.051^d^

^a^Geometric mean (95% CI), ^b^Student’s t test, ^c^Pearson’s Chi Square test, ^d^U of Mann Whitney, ^e^Fisher’s Exact Test, ^f^Basic Activities of Daily Living, ^g^Instrumental Activities of Daily Living, ^h^Physical Activity

The prevalence of dependence in IADL was 19%, while the prevalence of low physical performance was 17.33%.

In relation to the sociodemographic characteristics stratified by physical activity categories, we found that the participants who met the guidelines of PA were younger (69.5 years) in comparison to those who had low PA (< 3.0 METs) or where inactive (71.5 years). In addition, no statistically significant differences were observed across PA categories according to nutritional status, tobacco, drug, and alcohol consumption. The OA who met the guidelines of PA had fewer depressive symptoms, and better scores in BADL, IADL and SPPB test, compared to those who had low PA (< 3.0 METs) or where inactive (Table 2).

**Table 2:** Sociodemographic characteristics stratified by physical activity categories

	Low PA (< 3.0 METs) or inactive Mean ^a^ (IC 95%)	Insufficiently increased PA (≥ 3.0 < 6.0 METs) Mean ^a^ (IC 95%)	Met the guidelines (≥ 6 METs) Mean ^a^ (IC 95%)	*P* value
Age (years), mean (SD)	72.8 (8.3)	71.5 (7.5)	69.5 (7.4)	0.022 ^b^
Sex, women%	53 (94.6)	55 (87.1)	141 (77.9)	0.009
Marital status, n (%)				
Married	20 (23.2)	37 (44.0)	39 (44.3)	0.030 ^c^
Free Union	2 (2.3)	1 (1.1)	1 (1.1)	
Single	15 (17.4)	13 (15.4)	15 (17.0)	
Separated	1 (1.1)	6 (7.1)	3 (3.4)	
Divorced	8 (9.3)	2 (2.3)	4 (4.5)	
Widowed	40 (46.5)	25 (29.7)	26 (29.5)	
Educational level	8.2 (7.2, 9.3)	8.8 (7.8, 9.8)	10.1 (9.0, 11.2)	0.070 ^d^
Occupation, n (%)				
Unemployed	0 (0)	0 (0)	2 (2.2)	0.048 ^c^
Retiree with pension	21 (24.4)	27 (32.1)	25 (28.4)	
Retiree without pension	6 (6.9)	2 (2.3)	1 (1.1)	
Informal commerce	2 (2.3)	1 (1.1)	3 (3.4)	
Office worker	0 (0)	6 (7.1)	2 (2.2)	
Independent professional	4 (4.6)	0 (0)	5 (5.6)	
Businessman or boss	0 (0)	2 (2.3)	0 (0)	
Housewife	51 (59.3)	41 (48.8)	45 (51.1)	
Inactive	0 (0)	1 (1.1)	1 (1.1)	
Other activity	2 (2.3)	4 (4.7)	4 (4.5)	
Nutrition status, n (%)				
Normal	59 (74.6)	61 (74.3)	69 (80.2)	0.121 ^c^
Risk of malnutrition	17 (21.5)	21 (25.6)	17 (19.7)	
Malnutrition	3 (3.8)	0 (0)	0 (0)	
Tobacco consumption, n (%)				
Current tobacco use	10 (11.6)	13 (15.4)	10 (11.3)	0.440 ^e^
Currently does not consume	29 (33.7)	23 (27.3)	36 (40.9)	
Never	47 (54.6)	48 (57.1)	42 (47.7)	
Drugs, n (%)				
Consume	36 (41.8)	31 (37.3)	30 (34.4)	0.613 ^e^
Does not consume	50 (58.1)	52 (62.6)	57 (65.5)	
Alcohol, n (%)				
Has never drunk alcohol	30 (35.2)	22 (26.1)	26 (29.5)	0.254^c^
Currently does not drink	10 (11.7)	14 (16.6)	18 (20.4)	
Drink sporadically	41 (48.2)	40 (47.6)	38 (43.1)	
Less than once a week	2 (2.3)	2 (2.3)	5 (5.6)	
One or two days a week	1 (1.1)	2 (2.3)	0 (0)	
Five to six days a week	1 (1.1)	0 (0)	0 (0)	
Every day of the week	0 (0)	4 (4.7)	1 (1.1)	
Depressive symptoms	4.4 (3.6, 5.4)	4.6 (3.7, 5.7)	3.9 (3.1, 4.8)	0.702 ^d^
Cognitive status	26.4 (25.8, 27.1)	26.8 (26.1, 27.4)	26.4 (25.7, 27.2)	0.714 ^d^
Medications	4.4 (3.8, 5.2)	3.6 (3.1, 4.3)	4.2 (3.7, 4.8)	0.343 ^d^
Comorbidities	1.6 (1.4, 1.8)	1.7 (1.4, 1.9)	1.6 (1.4, 1.8)	0.741 ^d^
BADL^f^	97.5 (96.4, 98.7)	99.1 (98.6, 99.7)	98.6 (97.9, 99.4)	0.044 ^d^
IADL^g^	7.4 (7.1, 7.6)	7.6 (7.4, 7.8)	7.8 (7.7, 7.9)	0.016 ^d^
Physical performance	8.0 (7.5, 8.6)	9.0 (8.5, 9.4)	9.1 (8.7, 9.6)	0.005 ^d^

^a^ Geometric mean (95% CI), ^b^ ANOVA, ^c^ Pearson’s Chi Square, ^d^ U of Mann Whitney, ^e^ Fisher’s Exact Test, ^f^ Basic Activities of Daily Living, ^g^ Instrumental Activities of Daily Living

The association between moderate to vigorous PA and the BADL, IADL, and PP in OA are shown in Table 3. Moderate to vigorous PA was significantly associated with an increase of BADL (β = 0.19; 95% CI: 0.11, 0.26), IADL (β = 0.08; 95% CI: 0.05, 0.10) and PP (β = 0.10; 95% CI: 0.06, 0.13) scores.

**Table 3:** Association between moderate to vigorous physical activity and BADL and IADL and physical performance

	**BADL**^**d**^				**IADL**^**e**^				**Physical Performance**			
	Model 1^b^		Model 2^c^		Model 1^b^		Model 2^c^		Model 1^b^		Model 2^c^	
	ß^a^	CI 95%	ß^a^	CI 95%	ß^a^	CI 95%	ß^a^	CI 95%	ß^a^	CI 95%	ß^a^	CI 95%
**Moderate to vigorous PA (METs/day)**	0.17	(0.10, 0.24)	0.19	(0.11, 0.26)	0.05	(0.03, 0.07)	0.08	(0.05, 0.10)	0.06	(0.03, 0.08)	0.10	(0.06, 0.13)

^a^ Mixed effects multiple linear regression models

^b^ Model 1: Unadjusted model

^c^ Model 2: Adjusted by age, sex, occupation, marital status, educational level, nutritional status, comorbidities, medicine use, depressive symptoms cognitive level, tobacco, alcohol and drug use

^d^ Basic Activities of Daily Living

^e^ Instrumental Activities of Daily Living

Table 4 showed that OA who met the guidelines of PA (≥ 6 METs) had a lower probability of dependence in IADL (OR = 0.35; 95%CI: 0.13, 0.95 and OR = 0.17; 95%CI: 0.10, 0.80), and low PP (OR = 0.41; 95%CI: 0.19, 0.91 and OR = 0.18; 95%CI: 0.11, 0.58) in the adjusted models compared with those who had low PA (< 3.0 METs) or where inactive. No significant associations were found between PA and BADL.

**Table 4:** Risk of dependence for the BADL and IADL and physical performance according to the categories of physical activity

	**BADL**^**d**^				**IADL**^**e**^				**Physical Performance**			
	Model 1^b^		Model 2^c^		Model 1^b^		Model 2^c^		Model 1^b^		Model 2^c^	
	OR^a^	CI 95%	OR^a^	CI 95%	OR^a^	CI 95%	OR^a^	CI 95%	OR^a^	CI 95%	OR^a^	CI 95%
Low PA (< 3.0 METs) or inactive	1.0	–	1.0	–	1.0	–	1.0	–	1.0	–	1.0	–
Insufficiently increased PA (≥ 3.0 < 6.0 METs)	0.95	0.50, 1.70	0.96	0.51, 1.72	0.28	0.12, 0.63	0.35	0.13, 0.95	0.27	0.14, 0.54	0.41	0.19, 0.91
Met the PA guidelines (≥ 6 METs)	0.60	0.05, 6.74	0.84	0.13, 4.18	0.11	0.05, 0.37	0.17	0.10, 0.80	0.09	0.03, 0.25	0.18	0.11, 0.58

^a^ Generalized estimating equation (GEE) models

^b^ Model 1: Unadjusted model

^c^ Model 2: Adjusted by age, sex, occupation, marital status, educational level, nutritional status, comorbidities, medicine use, depressive symptoms, cognitive level, tobacco, alcohol and drug use

^d^ Basic Activities of Daily Living

^e^ Instrumental Activities of Daily Living

## Discussion

Our data suggests that OA who performed moderate to vigorous intensity PA, after 4.5 years of follow-up, had a lower risk of presenting dependence in IADL and a lower risk of having low PP. Therefore, our findings suggest that moderate to vigorous intensity PA is essential to maintain optimal functionality and good PP.

In relation to the association of PA and BADL, our study found that subjects in the highest category of PA, compared to those in the lowest category, had a 16% lower risk of dependence in BADL; however, this result was not statistically significant. According to the association of PA and BADL, previous studies [16, 45–48] have reported that PA reduces the risk of being dependent in BADL. For example, Stessman et al. [45] found that OA who performed PA at least 4 times a week preserved the ability to perform the BADL, both for women (OR = 8.5; 95%CI: 2.0, 36.4) and for men (OR = 4.3; 96%CI: 1.1, 17.1). Boyle et al. [46] observed that OA who perform 2.3 h a week of PA have a 16% lower risk of being dependent in BADL and a 41% lower risk for those who perform 7 h of PA a week. Additionally, Crevenna et al. [47] observed that low levels of PA (< 150 min/week) is associated with a higher risk of dependence in BADL (OR = 1.7; 95% CI 1.4, 2.2).

Performing PA increases muscular strength, which helps prevent dependency in ADL. People who have lower strength in the lower limb muscles, especially the hip extensors, have been reported to require more support for transfers, dressing, and going up and down stairs [48]. On the other hand, PA contributes to improving walking speed; this is important since this aspect has been seen to have a positive association with the execution of BADL [49].

In addition, we found that moderate to vigorous intensity PA is associated with a lower risk of dependence in the performance of IADL; this finding is consistent with what was mentioned in cohort studies. For example, Balzi et al. [16] reported that moderate to vigorous PA was associated with lower risk of dependence in IADL (OR -= 0.18; 95%CI: 0.09, 0.36) compared to low PA. A study from Boyle et al. [46] suggests that for each increase of one hour of PA per week, the risk of presenting dependence in IADL decreases by 7% (HR = 0.93; 95%CI: 0.89,0.98). This is because moderate to vigorous-intensity PA appears to have benefits on executive function, episodic memory, visuospatial function, verbal fluency, processing speed, and global cognitive function [50], which has an important impact on ADL. Some studies show that executive functions are relevant for preparing meals, taking medications, paying bills and planning the daily routine [51, 52]. In addition, PA improves strength and muscle mass and gait speed; these are important aspects to improve mobility and the execution of IADL [53].

On the other hand, we found that there is an association between moderate to vigorous intensity PA and physical performance (ß = 0.10, 95% CI 0.06, 0.13). This finding was consistent with that reported by previous studies such as the study carried out by Hsueh et al. [54], where it was reported that performing PA lasting 10 min is associated with better results in grip strength (β = 0.39; 95%CI: 0.12, 0.64) and in balance on one leg (β = 0.25; CI95%: 0.02, 0.49) in older women. Additionally, Morie et al. [55] mentions that doing physical activity is associated with better results in physical performance in the lower limbs (β = 1.13, *P* < 0.001). PA contributes to the maintenance of physical performance because it promotes changes in the musculoskeletal system, as it increases strength and muscle mass, prevents the infiltration of fat into muscle tissue, optimizes muscle regenerative capacity, prevents loss of bone mineral density, improves the mechanical and biological properties of the articular cartilage, the joint becomes more flexible and less fragile and also improves neural function, which is important to improve gait speed and balance [12, 56, 57].

Our results should be interpreted with caution because the present study has some limitations that should be considered. First, even though this is a cohort study, we cannot infer a cause-effect relationship. Second, the instruments used in this study have not had a cross-cultural adaptation and have not been validated in older Mexican adults living in the community, which can lead to information bias and misclassification. Nevertheless, these instruments have been validated in the Spanish-speaking population. Despite the fact that our analyses were adjusted for different confounding variables, residual confusion for unmeasured variables is possible, for example sarcopenia and muscle mass. A final limitation has to do with a possible selection bias, since the sample in our study was made up of volunteers who came on their own to the centers where the evaluations were carried out, and those older adults with the greatest impairment in physical or functional performance could have been excluded. The strengths of this study lie in the fact that robust statistical analyses were carried out to determine the association and the study follow-up period was long, which allowed associations to be detected between PA and physical and functional performance.

## Conclusion

This longitudinal study of OA that explores the association between physical activity over physical and functional performance, identified that the OA who realize PA of moderate to vigorous intensity was significantly associated with an increase of BADL (β = 0.19; 95% CI: 0.11, 0.26), IADL (β = 0.08; 95% CI: 0.05, 0.10) and PP (β = 0.10; 95% CI: 0.06, 0.13) scores. The OA that practice this intensity of PA presents less risk of dependence of BADL, IADL, and less risk of low physical performance. Our findings confirm that PA represents a cornerstone for a healthy ageing process and suggest they would keep or improve their functionality. From public health perspective, in Mexico is needed more strategies to promote the physical activities for older adults. Future research is needed to identify the complete dose of PA in community dwelling OA.

## Data Availability

The datasets used and/or analyzed during the current study are available from the corresponding author on reasonable request.

## Abbreviations

PA: Physical activity
OA: Older adults
FRAYSMEX: Frailty, Dynapenia and Sarcopenia in Mexican adults
CHAMPS: Community Healthy Activities Model Program for Seniors
SPPB: Short Physical Performance Battery
BADL: Basic activities of the daily life
IADL: Instrumental activities of the daily life
PP: Physical performance
OR: Odds ratio
95% CI: 95% Confidence interval
METs: Metabolic equivalents
MNA: Mini Nutritional Assessment
CESD-7: Center for Epidemiological Studies Depression Scale
